# Distinguishing features of acute Vogt-Koyanagi-Harada disease and acute central serous chorioretinopathy on optical coherence tomography angiography and en face optical coherence tomography imaging

**DOI:** 10.1186/s12348-016-0122-z

**Published:** 2017-01-13

**Authors:** Kanika Aggarwal, Aniruddha Agarwal, Ankit Deokar, Sarakshi Mahajan, Ramandeep Singh, Reema Bansal, Aman Sharma, Mangat R. Dogra, Vishali Gupta, for the OCTA Study Group

**Affiliations:** 1 Advanced Eye Center, Post Graduate Institute of Medical Education and Research, Sector 12, Chandigarh, 160012 India; 2 Ocular Imaging Research and Reading Center (OIRRC), Omaha, NE 68105 USA; 3 Department of Internal Medicine, Post Graduate Institute of Medical Education and Research, Sector 12, Chandigarh, 160012 India

**Keywords:** Central serous chorioretinopathy, EDI-OCT, Multimodal imaging, Optical coherence tomography angiography, Vogt-Koyanagi-Harada syndrome, Indocyanine green angiography

## Abstract

**Background:**

The aim of this study is to determine the differences in optical coherence tomography angiography (OCTA) features of acute Vogt-Koyanagi-Harada disease (VKH) and acute central serous chorioretinopathy (CSC). Clinical and imaging data of patients with acute CSC and VKH in a tertiary-care institute were analyzed. Multimodal imaging including fluorescein angiography, indocyanine green angiography (ICGA), and enhanced-depth imaging OCT were performed. OCTA images were analyzed for alterations in retinochoroidal microvasculature.

**Results:**

Thirty-four eyes (24 patients; 10 with VKH and 14 with CSC) were included. OCTA en face images showed apparent areas of choriocapillaris *flow void* due to shadowing effect from overlying subretinal fluid and pigment epithelial detachment in CSC. However, eyes with VKH showed presence of *true* choriocapillaris flow void on OCTA that corresponded to choriocapillaris ischemia on ICGA.

**Conclusions:**

OCTA is a useful tool to assess choriocapillaris ischemia in VKH and is helpful to differentiate it from CSC in the acute stage.

## Background

Vogt-Koyanagi-Harada disease (VKH) is a multisystem autoimmune disorder primarily affecting various pigmented tissues [1]. Ocular involvement in the acute stage of VKH is characterized by diffuse choroidal stromal inflammation and exudative retinal detachment [2]. The acute form of the disease is typically characterized by the presence of bilateral exudative retinal detachments. Such manifestations may also occur in acute central serous chorioretinopathy (CSC) leading to diagnostic dilemmas in certain situations. However, VKH is an inflammatory disease while the pathogenesis of CSC is mainly attributed to choroidal vascular dysfunction [3–6]. CSC may often be misdiagnosed as VKH [7, 8], particularly when it presents with bilateral serous retinal detachment and increased choroidal thickness on enhanced depth imaging optical coherence tomography (EDI-OCT) [9]. Failure in differentiating CSC from VKH may result in inappropriate treatment with corticosteroids or delay in therapy and irreversible visual loss.

The diagnosis of both VKH and CSC is based on a number of factors such as history and clinical examination combined with findings on retinal and choroidal imaging on OCT, fluorescein angiography (FA), and indocyanine green angiography (ICGA). Conventionally, ICGA and FA are useful in detecting characteristic abnormalities such as early choroidal stromal hyperfluorescence, hypofluorescent dark dots and early pinpoint hyperfluorescence with late pooling of dye in VKH, and choroidal hyperfluorescence and focal leaks with a smokestack or inkblot pattern in CSC [10–12]. However, these imaging modalities are invasive with a risk of dye-related serious adverse reactions. Moreover, while they may help in distinguishing the two conditions, the dye-based techniques are unable to provide detailed assessment of microstructural vascular alterations in the deep retinal plexus, retinal pigment epithelium (RPE), and the choriocapillaris.

More recently, optical coherence tomography angiography (OCTA) has emerged as a useful non-invasive imaging technique which provides a detailed depth-resolved reconstruction of the retinochoroidal microvasculature by utilizing endoluminal flow as intrinsic contrast [13]. Thus, it may provide novel insights into the underlying pathophysiologic mechanisms in both CSC and VKH. OCTA has shown to be a sensitive tool in detecting abnormalities such as choroidal neovascularization in CSC [14, 15] and dynamic changes in the choriocapillaris layer in the form of multiple dark areas of choriocapillaris loss suggestive of “flow void” in VKH. The index study aims to compare the OCTA findings in the acute stages of CSC and VKH disease and to determine its role in differentiating the two conditions.

## Methods

The index study was approved by the Institutional Review Board/Ethics Committee (IEC) of the Post Graduate Institute of Medical Education and Research (PGIMER), Chandigarh, India. Informed consent was obtained from all individual participants included in the study. Imaging data of patients with acute CSC and VKH disease attending the Retina and Uveitis services of Advanced Eye Center, PGIMER, were reviewed. The study adhered to the tenets of the Declaration of Helsinki and the rules laid down by Health Insurance Portability and Accountability Act (HIPAA) of 1996.

Consecutive subjects with the diagnosis of acute CSC and VKH between June 2015 and January 2016 were enrolled in the study. The diagnosis of VKH was based on the revised criteria by the First International Workshop on Vogt-Koyanagi-Harada Disease, 2001 [1]. These criteria are based on history, systemic examination, and ocular features on indirect ophthalmoscopy and imaging such as FA or ultrasonography. The diagnosis of CSC was based on clinical and multimodal imaging results such as FA findings of idiopathic focal leakage at the level of the RPE, and serous retinal detachment on optical coherence tomography [6]. Patients diagnosed with other confounding retinal pathologies such as age-related macular degeneration, idiopathic choroidal neovascularization, polypoidal choroidal vasculopathy, diabetic retinopathy, glaucoma, or optic neuropathy were excluded from the study.

Patients fulfilling the above diagnostic criteria and undergoing OCTA using the Optovue RTVue XR 100 Avanti (Optovue Inc., Fremont, CA, USA) were included in the study. This device is a spectral-domain-based OCT which uses the technology of split-spectrum amplitude decorrelation algorithm (SSADA) for the acquisition of OCTA images. The device has an A-scan rate of 70,000 per second and uses a light source centered on 840 nm to obtain 3 × 3 mm, 6 × 6 mm, or 8 × 8 mm OCTA images. The device co-registers the en face OCTA images with the cross-sectional OCT B-scans which allows point-to-point correlation between the two. In addition, the software provides structural en face images so that the details of the vasculature can be analyzed keeping the signal strength in consideration. OCTA images were analyzed using the RTVue software. This software provides automatic segmentation to analyze the superficial and deep retinal vascular plexus, outer retina, and choriocapillaris. However, the OCTA and OCT B-scans were manually scrolled to confirm the findings and detect projection and other artifacts.

Combined FA and ICGA images were obtained using Heidelberg Spectralis® (Heidelberg Engineering, Heidelberg, Germany) for patients with VKH, and FA images were obtained for patients with CSC. All the patients also underwent SD-OCT imaging with Spectralis® (Heidelberg Engineering, Germany) with the EDI function activated. Color fundus photography performed using ultra-wide field retinal camera (Optos P200Tx, Optos Inc., Scotland, UK) and/or conventional fundus camera (Carl Zeiss FF450, Zeiss Meditec, La Jolla, CA, USA) was available for analyses for all the patients included in the study.

Two trained masked graders (A.A. and K.A.; ophthalmologists with subspecialty training in retina and uveitis) performed all the image analyses in the study independently. Any disagreements between the graders were resolved with open adjudication. En face OCTA and structural en face images were analyzed to assess alterations in the retinochoroidal vasculature. These findings were compared to other imaging techniques to confirm changes such as decrease or absence of blood flow and anatomical changes in the microvasculature.

## Results

The study included 14 subjects diagnosed with CSC (2 females) with a mean age of 34.57 ± 5.38 years. Ten subjects (7 females) were diagnosed with VKH disease (mean age 29.9 ± 14.02 years). All subjects included in the study were Asian Indians. All the subjects with acute CSC had subretinal fluid in at least one eye at the time of presentation. Among subjects with VKH, 14 out of 20 eyes showed presence of subretinal fluid at the time of initial presentation. Eight patients with CSC had a history of systemic corticosteroid exposure. Clinical and demographic details of all the subjects are listed in Tables 1 and 2.

**Table 1:** Demographic data and clinical features of patients with acute central serous chorioretinopathy included in the study

Patient no.	Sex	Age (years)	Eye	BCVA	Anterior segment features	Posterior segment features	Systemic features and steroid exposure	Total follow-up (weeks)
1	M	32	OD	0.8	–	SSRD and PED	Nil	6
			OS	0.2		–		
2	M	41	OD	0	–	–	Nil	12
			OS	0.2		SSRD		
3	M	33	OD	0.2	–	–	Local steroid use for dermatological condition	8
			OS	0.8		SSRD		
4	F	38	OD	0	–	–	Oral herbal medications for hypertension	2
			OS	0.2		SSRD and PED		
5	M	37	OD	0.8	–	SSRD	Oral herbal medications for renal calculi	8
			OS	0		–		
6	M	32	OD	0.2	–	–	Local steroid use for tinea	4
			OS	0.48		SSRD		
7	M	35	OD	0	–	SSRD and PED	Oral steroids for asthma	6
			OS	0		–		
8	M	32	OD	0	–	PED	Nil	12
			OS	0.48		SSRD and PED		
9	M	27	OD	0	–	–	Nil	14
			OS	0.3		SSRD and PED		
10	M	32	OD	0.2	–	SSRD	Nil	9
			OS	0		–		
11	F	24	OD	0.1	–	SSRD and PED	Local steroid use for dermatological condition	5
			OS	0.1		–		
12	M	44	OD	0.3	–	SSRD	Oral steroids for joint pains	10
			OS	0		–		
13	M	38	OD	0	–	–	Nil	12
			OS	0.1		SSRD and PED		
14	M	39	OD	0.2	–	SSRD and PED	Oral herbal medications for fatigue	4
			OS	0		–		

*BCVA* best-corrected visual acuity, *F* female, *M* male, *OD* right eye, *OS* left eye, *PED* pigment epithelial detachment, *SSRD* subfoveal serous retinal detachment

**Table 2:** Demographic data and clinical features of patients with acute Vogt-Koyanagi-Harada disease included in the study

Patient no.	Sex	Age (years)	Eye	BCVA	Anterior segment features	Posterior segment features	Systemic features	Total follow-up (weeks)
1	M	36	OD	0	No cells/flare/KPs	–	–	5
			OS	1	No cells/flare/KPs	Disc edema; SSRD		
2	F	39	OD	0.8	No cells/flare/KPs	Disc edema	Headache	3
			OS	0.8	No cells/flare/KPs	SSRD; multiple choroiditis lesions		
3	F	27	OD	0	No cells/flare/KPs	Chorioretinal scars	Headache and fever	13
			OS	0.6	Cells 0.5+, pigment over anterior lens surface	Multiple choroiditis lesions; SSRD		
4	M	33	OD	0.8	Cells 1+, pigment over anterior lens surface	Vitritis, disc edema; SSRD	–	14
			OS	0.8	No cells/flare/KPs	Disc edema; SSRD		
5	F	19	OD	0	No cells/flare/KPs	–	Headache	14
			OS	1	No cells/flare/KPs	Multiple choroiditis lesions		
6	F	60	OD	HMCF	Cells 3+, flare +	Vitritis; increased choroidal thickness	Headache; sensorineural hearing loss +	1
			OS	1	Cells 2+, flare +	Vitritis; increased choroidal thickness		
7	M	19	OD	0	No cells/flare/KPs	Multiple choroiditis lesions and SSRD	–	41
			OS	0.9	No cells/flare/KPs	Multiple choroiditis lesions and SSRD		
8	F	32	OD	1	No cells/flare/KPs	Disc edema SSRD; increased choroidal thickness	Headache	5
			OS	0.1	No cells/flare/KPs	Multiple choroiditis lesions		
9	F	5	OD	0.8	No cells/flare/KPs	SSRD; increased choroidal thickness	–	12
			OS	0.8	No cells/flare/KPs	SSRD; vitritis; increased choroidal thickness		
10	F	28	OD	0.2	No cells/flare/KPs	SSRD; increased choroidal thickness	Headache	10
			OS	0.48	No cells/flare/KPs	SSRD; increased choroidal thickness		

*BCVA* best-corrected visual acuity, *F* female, *M* male, *OD* right eye, *OS* left eye

The mean subfoveal choroidal thickness was 342.29 ± 44.89 μm in patients with acute CSC and 407 ± 114 μm in eyes with acute VKH disease.

### Findings on OCTA in CSC

**Fig. 1:**
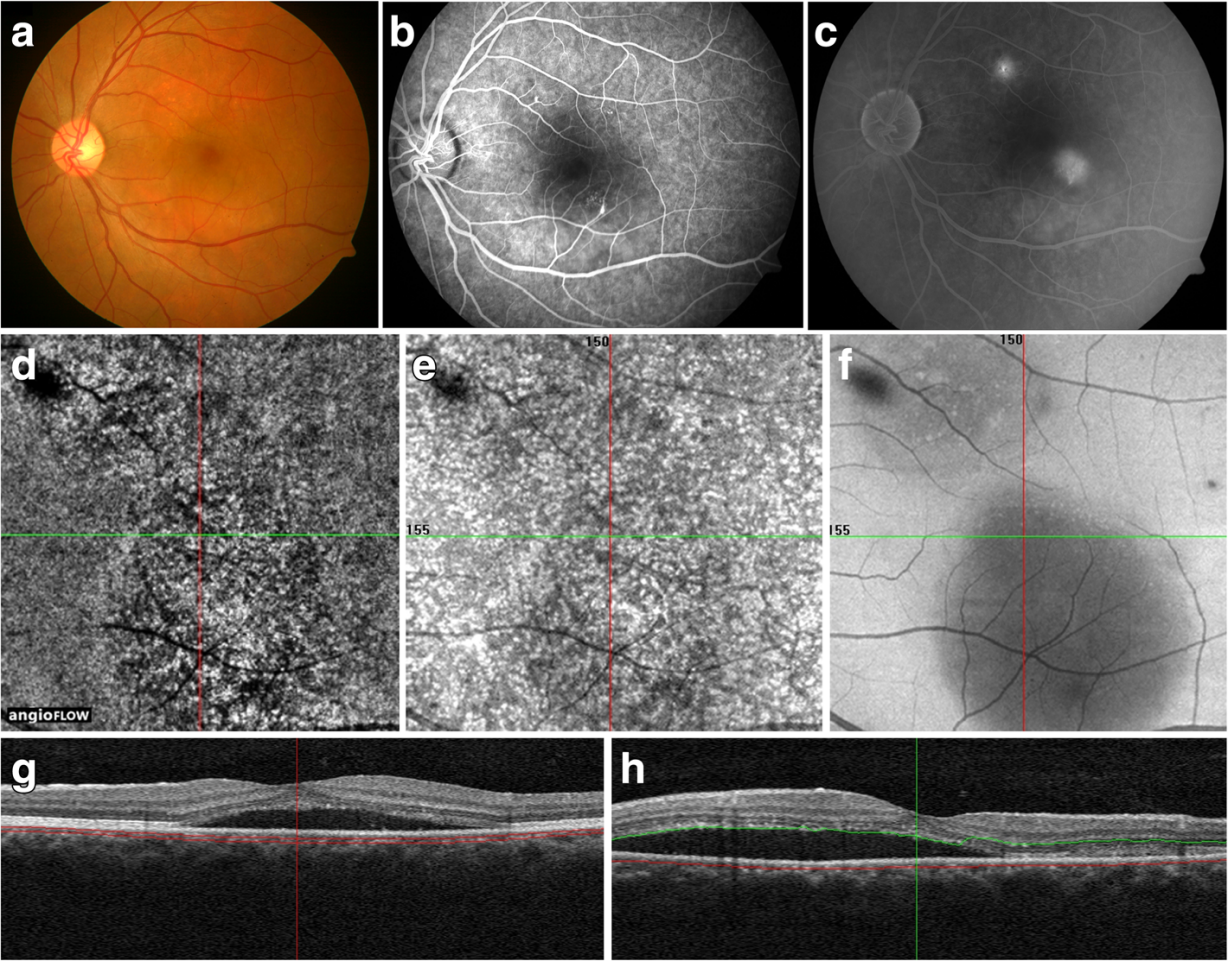
Multimodal imaging of a patient with acute central serous chorioretinopathy (CSC) (subject #6). Fundus photograph (**a**) of the left eye shows presence of subretinal fluid (SRF) in the macula. Fluorescein angiography (FA) in the early phase (**b**) shows pin-point hyperfluorescence superonasal and inferotemporal to the fovea. The late phase FA (**c**) shows *expanding dot* sign with pooling of the dye. The en face optical coherence tomography angiography (OCTA) (**d**) shows the presence of *mottled dark areas* which corresponded to the dark areas representing signal loss on the corresponding structural en face OCT at the level of the choriocapillaris (**e**) and the outer retina (**f**). *Horizontal* (**g**) and *vertical* (**h**) cross-sectional OCT B-scan shows the presence of SRF in the macula

The OCTA images in patients with acute CSC at the level of the superficial and deep retinal capillary plexus did not show any significant vascular abnormalities. Similarly, the OCTA images at the avascular outer retinal slab did not show any abnormalities such as neovascular membranes. At the level of the choriocapillaris, en face OCTA images showed two patterns of alterations: (1) *mottled dark areas* and (2) *dense dark areas* of *possible* flow void (Table 3). Mottled dark areas appeared as irregular hyporeflective areas on en face OCTA in 14 eyes (50%) (Fig. 1). On comparing the OCTA images with corresponding OCT B-scans, these mottled dark areas were co-localized with subretinal fluid. The corresponding structural en face OCT scan also showed presence of shadowing due to loss of signal transmission in the areas of apparent flow void. Thus, these dark areas were likely due to *signal loss* rather than true choriocapillaris flow void (Fig. 1).

**Table 3:** Multimodal imaging features of alterations observed on optical coherence tomography angiography in patients with acute Vogt-Koyanagi-Harada syndrome and acute central serous chorioretinopathy

Feature	Condition	Appearance of choriocapillaris on OCTA	Associated retinal/pigment epithelial/choroidal features	Features on EDI-OCT and/or ICGA
Dense dark areas	CSC	Single well-defined uniformly hyporeflective areas of apparent flow void	PED	Hyper-reflective dome-shaped elevation of the RPE on EDI-OCT
Mottled dark areas	CSC	Irregular, areas of mixed hypo- and hyper-reflectance with ill-defined margins	SSRD	Homogenous hyporeflective space between the neurosensory retina and RPE on EDI-OCT
Multifocal dark spots	VKH	Multiple, round-to-oval, well-defined, variably sized hyporeflective areas suggestive of choriocapillaris flow void	Choroidal inflammatory foci	Choriocapillaris ischemia on ICGA

**Fig. 2:**
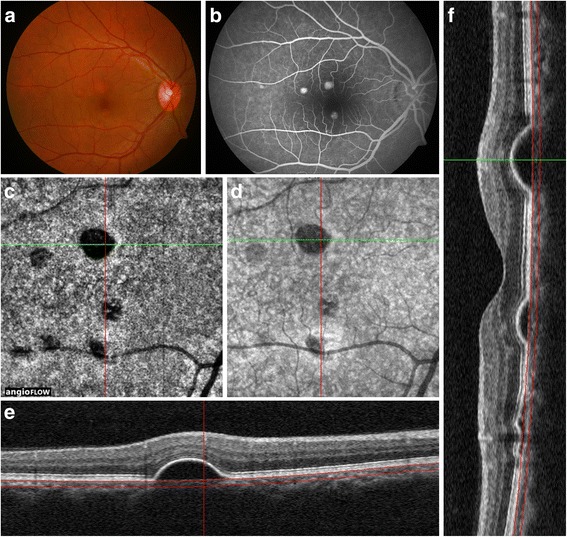
Multimodal imaging of a patient with acute central serous chorioretinopathy (CSC) (subject #8). Fundus photograph of the right eye (**a**) shows presence of multiple pigment epithelial detachments (PEDs) in the macula. (**b**) On fluorescein angiography (FA), there is hyperfluorescence corresponding to the PEDs. (**c**) The en face optical coherence tomography angiography (OCTA) shows presence of *dense dark areas* at the level of the choriocapillaris. (**d**) The structural en face OCT shows hyporeflectance suggestive of loss of signal transmission in the areas of PEDs. The *horizontal* (**e**) and *vertical* (**f**) cross-sectional OCT B-scans show dome-shaped PEDs

In nine eyes (32.14%), there were multiple, hyporeflective, round-to-oval, dense dark areas corresponding to the pigment epithelial detachments (PED) observed on OCT B-scans (Fig. 2). The structural en face OCTs also showed complete loss of signal transmission in these areas of PED. Thus, dense dark areas on OCTA in CSC were also unlikely to represent true choriocapillaris hypoperfusion.

### Findings on OCTA in VKH

**Fig. 3:**
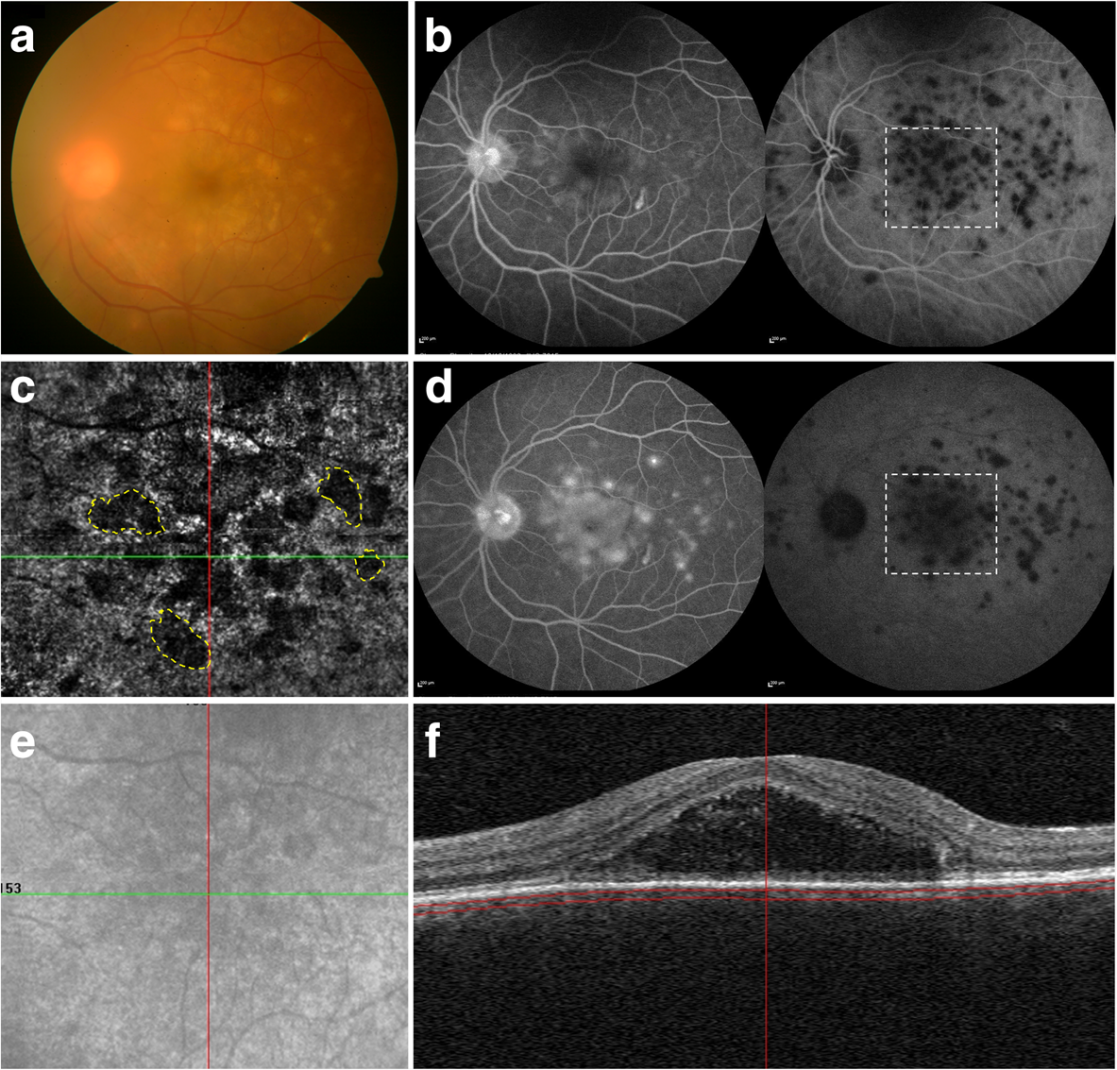
Fundus photography, combined fluorescein angiography (FA) and indocyanine green angiography (ICGA), and optical coherence tomography angiography (OCTA) of a patient with acute Vogt-Koyanagi-Harada (VKH) disease (subject #3). Fundus photograph (**a**) shows presence of vitritis with multiple yellowish choroiditis lesions in the posterior pole. Combined FA and ICGA in the early frame (**b**) shows early pin-point hyperfluorescence on FA and multiple hypocyanescent lesions on ICGA. In the late frame (**d**), there is progressive hyperfluorescence with pooling of the dye on FA and persistence of hypocyanescent lesions on ICGA suggestive of choriocapillaris ischemia. The en face OCTA (**c**) at the level of choriocapillaris shows presence of *multifocal dark spots* which are variably sized and corresponded to the hypocyanescent lesions on ICGA. Few such dark foci have been demarcated with *yellow dashed line*. The structural en face OCT scan shows only mild signal loss in the area of subretinal fluid but no loss in the areas of *dark spots* (**e**). The corresponding cross-sectional OCT B-scan (**f**) shows presence of subretinal fluid

The superficial and deep retinal capillary plexuses did not show any significant changes on OCTA in patients with acute VKH disease. The avascular outer retinal slab on OCTA did not reveal presence of any abnormalities except signal loss. The en face OCTA scans at the level of the choriocapillaris showed multiple, discrete, dark areas which were variable in shape and size and widespread in distribution (20 eyes; 100%). Unlike CSC, these *multifocal dark spots* were not co-localized to the area of subretinal fluid but rather present throughout the area scanned by OCTA (Table 3; Fig. 3). The corresponding structural en face and cross-sectional OCTA images did not show any loss of signal transmission in these dark areas indicating that these may likely represent true flow void. The EDI-OCT scan passing through these hyporeflective areas showed features suggestive of choriocapillaris ischemia in these eyes.

Among eyes with VKH disease, ICGA imaging showed characteristic multiple hypofluorescent lesions in the early phase which remained hypofluorescent till the late phase suggestive of choriocapillaris ischemia (Fig. 3). These hypofluorescent spots showed a consistent correlation with the dark areas of flow void seen on OCTA scans at the level of choriocapillaris in all study subjects with VKH.

**Fig. 4:**
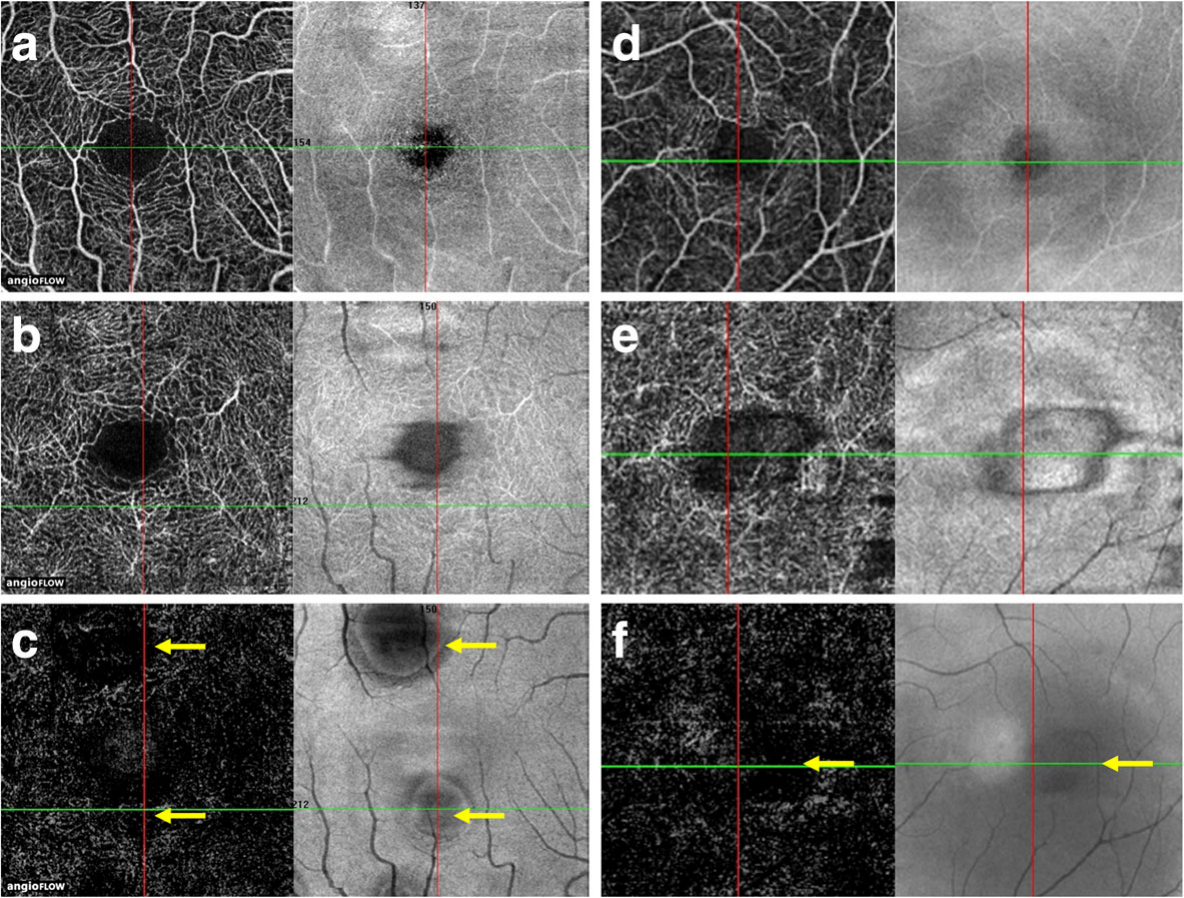
Comparison between the superficial and deep retinal plexus (SCP and DCP) and outer retinal slab of optical coherence tomography angiography (OCTA) of patients with central serous chorioretinopathy (CSC) (**a**–**c**) and Vogt-Koyanagi-Harada (VKH) disease (**d**–**f**). The OCTA image and the corresponding structural en face image at the SCP and DCP of patients with CSC and VKH (**a**, **b**, **d**, and **e**) do not show any abnormalities. The avascular outer retinal slab in CSC and VKH (**c**, **f**) shows few dark areas (*yellow arrows*) due to the signal loss from subretinal fluid as visualized in the corresponding structural en face OCT

A comparison of OCTA findings with clinical and multimodal imaging features in acute VKH and CSC is provided in Table 3. Figure 4 compares the findings of OCTA in the superficial and deep retinal plexus and the outer retinal slab of patients with VKH and CSC.

## Discussion

Bilateral intraocular inflammation with exudative retinal detachment is a hallmark clinical feature of VKH disease in the acute stage [1, 2, 4, 5]. However, this entity may be misdiagnosed as acute CSC, particularly in cases presenting with bilateral neurosensory detachments and PEDs [7, 8]. The pathophysiology of the two conditions is different since VKH is primarily a diffuse stromal choroiditis with secondary involvement of the choriocapillaris, whereas as CSC occurs a result of choroidal vascular hyperpermeability. Typically, these entities can be differentiated on the basis of history, clinical features, and ancillary investigations like FA, ICGA, and EDI-OCT. In a series reported by Yang et al. [16], 22% of patients with VKH were initially misdiagnosed as CSC. Another study by Shin et al. [7] reports 14.3% of VKH cases misdiagnosed due to absence of cellular inflammatory reaction or typical features such as sub-retinal septae and RPE folds on EDI-OCT in these eyes.

In the index study, we have described the differentiating features of VKH and CSC in the acute stage using the non-invasive technique of OCTA and en face OCT. OCTA has been recently shown to be an effective tool to identify the changes at the level of choriocapillaris in acute VKH and in monitoring the disease resolution, recurrence, and persistence in conjunction with clinical examination and other multimodal imaging techniques by our group [17]. In the present study, en face OCTA images in the acute stage of VKH showed multiple dark foci of flow void suggestive of choriocapillaris hypoperfusion which were variable in shape and size (20 eyes; 100%). ICGA imaging in these eyes confirmed the presence of choriocapillaris ischemia in the areas of choroidal inflammatory foci. The corresponding OCTA structural en face images showed no evidence of signal loss suggesting that these hyporeflective areas represented true flow void (Fig. 3).

In contrast with VKH disease, eyes with acute CSC showed two patterns of alterations on en face OCTA imaging and en face OCT scans. One pattern, mottled dark areas appeared as irregular dark areas on OCTA that corresponded to the subretinal fluid on OCT B-scan. The second pattern of alteration resulted due to RPE detachments and was seen as hyporeflective, dense dark areas (Table 3). The structural en face images showed signal loss due to the shadowing effect of the overlying retinal structures, subretinal fluid, and RPE, thereby confirming that such dark hyporeflective areas did not represent true flow void.

In a recently published study on OCTA in acute CSC, Costanzo et al. [18] described similar “dark areas,” “dark spots,” and “abnormal choroidal vessel pattern” on en face OCTA images at the level of choriocapillaris. The authors described dark areas that corresponded to areas of serous retinal detachment. These areas were attributed to either signal loss due to subretinal fluid, PED, outer segment photoreceptor elongation, or due to focal atrophy of the choriocapillaris secondary to compression by the enlarged vessels from the outer choroid. However, in our study, we have clearly demonstrated using multimodal imaging and analysis of the structural en face images that such areas of apparent flow void which appear as hyporeflective dark irregular areas (14 eyes; 50%) are due to signal attenuation from overlying structures (Fig. 1). In the study by Costanzo et al. [18], the dark spots were attributed to focal flow reduction in the choriocapillaris. However, in our study, such dark spots were observed in nine eye (32.14% eyes) areas of overlying PEDs without any evidence of choriocapillaris flow reduction. The *abnormal choroidal vessels* referred to dilated choroidal vessels showing typical or indistinct “tangled pattern” or “pruned tree pattern.” However, we did not note any such abnormal pattern of choroidal vessels in our patients. This could be due to the fact that we included only patients with acute CSC in our study whereas choroidal neovascularization is mainly a feature of chronic CSC.

Another study performed in patients with acute CSC by Feucht et al. [19] has described dark areas on en face OCTA scans at the level of choriocapillaris as “avascular” areas. The authors correlated these areas with other imaging techniques to identify if such dark areas represent detached retina. The results of the study suggested that such dark areas may be due to unexplained abnormal blood flow through choriocapillaris in some eyes. This is in contrast to our study where such areas of mottled appearance of hypo- and hyper-reflectance on OCTA en face and structural en face images were due to irregular loss of signal transmission and not due to focal choriocapillaris ischemia or hypoperfusion as suggested in the previous studies.

An important aspect of the index study is that among patients where the diagnosis of acute VKH disease versus acute CSC is being considered, non-invasive technique of OCTA and en face OCT may greatly aid in determining the accurate diagnosis. In eyes with either or both patterns of mottled dark areas and dense dark areas, the diagnosis of acute CSC is more likely since this condition is not associated with true choriocapillaris flow void. On the other hand, if the OCTA images show presence of variably sized round-to-oval multifocal dark spots that show no signal loss on structural en face OCT, true choriocapillaris ischemia is likely, thus favoring the diagnosis of acute VKH disease. Such an imaging analysis may avoid the need of invasive imaging techniques such as ICGA which may be more expensive or associated with systemic risks of allergies and side effects.

The limitations of our study are that it is a cross-sectional study and the sample size is modest. The technique of OCTA has a number of limitations such as presence of several artifacts in OCTA such as signal loss which need to be carefully interpreted in conjunction with multimodal imaging techniques [20]. Moreover, we included only patients with acute disease in our cohort. Thus, chronic changes such as choroidal neovascularization could not be assessed in our study.

## Conclusions

In summary, the diagnosis of acute VKH and CSC may pose a clinical challenge. While accurate diagnosis can be established in most cases on the basis of meticulous clinical examination and ancillary imaging, few cases may have overlapping features making the diagnosis and initiation of therapy difficult. In such clinical scenarios, OCTA imaging may have a role to aid in the diagnosis by identifying features of true choriocapillaris ischemia in eyes with acute VKH disease and absence of such ischemic changes in acute CSC, rapidly and non-invasively.

## References

[1] Read RW, Holland GN, Rao NA (2001). Revised diagnostic criteria for Vogt-Koyanagi-Harada disease: report of an international committee on nomenclature. Am J Ophthalmol.

[2] Rao NA, Gupta A, Dustin L (2010). Frequency of distinguishing clinical features in Vogt-Koyanagi-Harada disease. Ophthalmology.

[3] Prunte C, Flammer J (1996). Choroidal capillary and venous congestion in central serous chorioretinopathy. Am J Ophthalmol.

[4] Fang W, Yang P (2008). Vogt-koyanagi-harada syndrome. Curr Eye Res.

[5] Read RW (2002). Vogt-Koyanagi-Harada disease. Ophthalmol Clin North Am.

[6] Yannuzzi LA (2010). Central serous chorioretinopathy: a personal perspective. Am J Ophthalmol.

[7] Shin WB, Kim MK, Lee CS, Lee SC, Kim H (2015). Comparison of the clinical manifestations between Acute Vogt-Koyanagi-Harada disease and acute bilateral central serous chorioretinopathy. Korean J Ophthalmol.

[8] Lin D, Chen W, Zhang G (2014). Comparison of the optical coherence tomographic characters between acute Vogt-Koyanagi-Harada disease and acute central serous chorioretinopathy. BMC Ophthalmol.

[9] Imamura Y, Fujiwara T, Margolis R, Spaide RF (2009). Enhanced depth imaging optical coherence tomography of the choroid in central serous chorioretinopathy. Retina (Philadelphia, Pa).

[10] Menchini U, Virgili G, Lanzetta P, Ferrari E (1997). Indocyanine green angiography in central serous chorioretinopathy. ICG angiography in CSC. Int Ophthalmol.

[11] Piccolino FC, Borgia L, Zinicola E, Zingirian M (1995). Indocyanine green angiographic findings in central serous chorioretinopathy. Eye (Lond).

[12] Fardeau C, Tran TH, Gharbi B, Cassoux N, Bodaghi B, LeHoang P (2007). Retinal fluorescein and indocyanine green angiography and optical coherence tomography in successive stages of Vogt-Koyanagi-Harada disease. Int Ophthalmol.

[13] Agrawal R, Xin W, Keane PA, Chhablani J, Agarwal A (2016). Optical coherence tomography angiography: a non-invasive tool to image end-arterial system. Expert Rev Med Devices.

[14] Bonini Filho MA, de Carlo TE, Ferrara D (2015). Association of choroidal neovascularization and central serous chorioretinopathy with optical coherence tomography angiography. JAMA Ophthalmology.

[15] Quaranta-El Maftouhi M, El Maftouhi A, Eandi CM (2015). Chronic central serous chorioretinopathy imaged by optical coherence tomographic angiography. Am J Ophthalmol.

[16] Yang P, Ren Y, Li B, Fang W, Meng Q, Kijlstra A (2007). Clinical characteristics of Vogt-Koyanagi-Harada syndrome in Chinese patients. Ophthalmology.

[17] Aggarwal K, Agarwal A, Mahajan S (2016). The Role of optical coherence tomography angiography in the diagnosis and management of acute Vogt-Koyanagi-Harada disease. Ocul Immunol Inflamm.

[18] Costanzo E, Cohen SY, Miere A (2015). Optical coherence tomography angiography in central serous chorioretinopathy. J. Ophthalmol..

[19] Feucht N, Maier M, Lohmann CP, Reznicek L (2016). OCT angiography findings in acute central serous chorioretinopathy. Ophthalmic Surg Lasers Imaging Retina..

[20] Spaide RF, Fujimoto JG, Waheed NK (2015). Image artifacts in optical coherence tomography angiography. Retina (Philadelphia, Pa).

